# The Proper Housing of Incurables

**Published:** 1907-08-24

**Authors:** 


### HOSPITAL ADMINISTRATION.

### CONSTRUCTION AND ECONOMICS.

### THE PROPER HOUSING OF INCURABLES.

HOME AND HOSPITAL FOR JEWISH INCURABLES, TOTTENHAM.

We have on more than one occasion called attention to serious defects which exist in connection with the management and administration of the affairs of hospitals for incurables in this country. We recently raised our voice in protest against the too lavish expenditure which is being incurred by certain authorities in regard to the provision of open-air blocks for the reception of incurable maniacs resident in asylums. There is, however, a wide gap between unnecessary and indefensible extravagance on expenditure, and an outlay upon upkeep, nursing care and maintenance, justified by the efficiency of the arrangements and the comfort of the patients. The case, too, of those who, though incurably ill, have a clear and intelligent mind, apart altogether from frequent and sometimes continuous suffering, presents one of the saddest pictures known to man. It is clearly the duty and privilege of the hale to take especial pains to provide and secure adequate care for incurable cases of this latter class. Yet we have formed the opinion from our inspection of various institutions that it is rare to find a high tone and the absence of a despotic spirit within the hospitals for incurables with which we are acquainted. We suppose that the very fact that patients may reside in such a hospital for decades of years tends to create a certain atmosphere of discontent, which is not unnaturally resented by the officials. So a despotic spirit may be found in antagonism to something akin to a sense of oppression among the patients. Whatever be the cause it is clear to us that it would be a great blessing if the spirit of real genuine kindness, forbearance and patience could be maintained within the walls of every institution devoted to the care of incurable patients. At present this spirit is sometimes far to seek, and to its absence may be due a multitude of small miseries which in the aggregate may mean much to individual patients during the long martyrdom of suffering which patients have to undergo as a qualification for admission.

Quite recently the Chief Rabbi very kindly and insistently took us to see the Home and Hospital for Jewish Incurables, High Road, South Tottenham. This institution, after seventeen years of existence, has found an excellent site at Tottenham, and has been designed upon a plan which makes it more than usually attractive as a place of residence. There are a sufficiency of gardens laid out in an attractive manner to make it possible to give the 66 inmates all the pleasure which open-air life affords at such seasons as it may be a pleasurable one in these islands. Of the 66 inmates, 28 are men and 38 women; the care of these patients is entrusted to a staff of nine female and four male nurses, in addition to the matron and assistant matron, and there are nine domestic servants including the cook, with an engineer, a porter and a gardener also employed. In addition to a consulting staff there are also a medical officer and a dental surgeon. The expenditure in 1906 amounted to £3,852, of which sum the income fell short by upwards of £900. The Chief Rabbi, who is the patron of this institution, pays it periodical visits, takes the deepest interest in its welfare, and never fails to conduct a service on each occasion. We may further note that the president is Mr. Stuart M. Samuel, M.P., whose services to the institution were recorded on his fiftieth birthday by a resolution of the Board of Management, which declares that, the institution is under a deep debt of gratitude to this gentleman for his powerful advocacy of its case, as well as for the many years of service he has ungrudgingly devoted to its interests. Certainly Mr. Samuel may be proud to be president of so fine a building, which is maintained in excellent order by Miss Olga Phillip, the matron, and her staff, and which has an atmosphere about it which conveys the impression that here at any rate every patient is comfortable and happy. We have never visited any home for incurables where we have felt so satisfied that this was the case. We hope, however, that the demands for admission will not lead the Board to crowd the wards, for, should overcrowding be permitted, much of the usefulness and more of the comfort of the patients may be destroyed, whilst the health of all the inmates may be seriously affected for the worse.

No portion of the community provides so carefully, and on the whole so well for its poorer members as the Hebrew race, and we feel confident that if, as the report records, the applicants awaiting admission attain to a number which will necessitate the completion of the entire building, according to the original plans, prompt steps will be taken to issue an appeal. We have every confidence that all the money needed will speedily be subscribed, for this institution, as its name implies, is not only a hospital, but, as far as we can judge, a home in the best meaning of that word. Entering the institution as we did, without notice, and having gone carefully over it from top to bottom, we formed the conclusion that the general management was good and sound. We incline to the feeling, however, that more active supervision might be exercised over the sanitary sections of that portion of the building which is devoted to male patients. Here in all institutions of the kind constant, intelligent, self-denying supervision is essential, for without it the atmosphere of the wards and corridors may suffer, and, in addition, not only the health of all but the reputation of those immediately responsible. We mention this point because it brings out clearly that as a whole the Home and Hospital for Jewish Incurables at Tottenham is an institution of which its conductors may feel deservedly proud. We hope that it will long continue its useful work, and that its funds may always be at least adequate to meet the claims and necessities of every deserving incurable.

